# Supplementary material for: Governance quality indicators for organ procurement policies
Source: PLoS One. 2021 Jun 4;16(6):e0252686. doi: 10.1371/journal.pone.0252686 (PMC8177644; doi:10.1371/journal.pone.0252686)
Supplement: S1 File — (DOCX) [file pone.0252686.s001.docx]

**S1 File**

Content:

1. Fig A. *Health Governance Indicator I*
2. Fig B. *Health Governance Indicator II: Fulfilment/contravention of unexpressed preferences and policy awareness*
3. Table 1. Sampling frame
4. Participants’ information sheet
5. Survey instrument. *International Student Survey about Knowledge and Attitudes about Donation and Transplantation of Organs/Body Parts (ISSATO)*
6. **Fig A. *Health Governance Indicator I: Policy support and policy knowledge in each country***


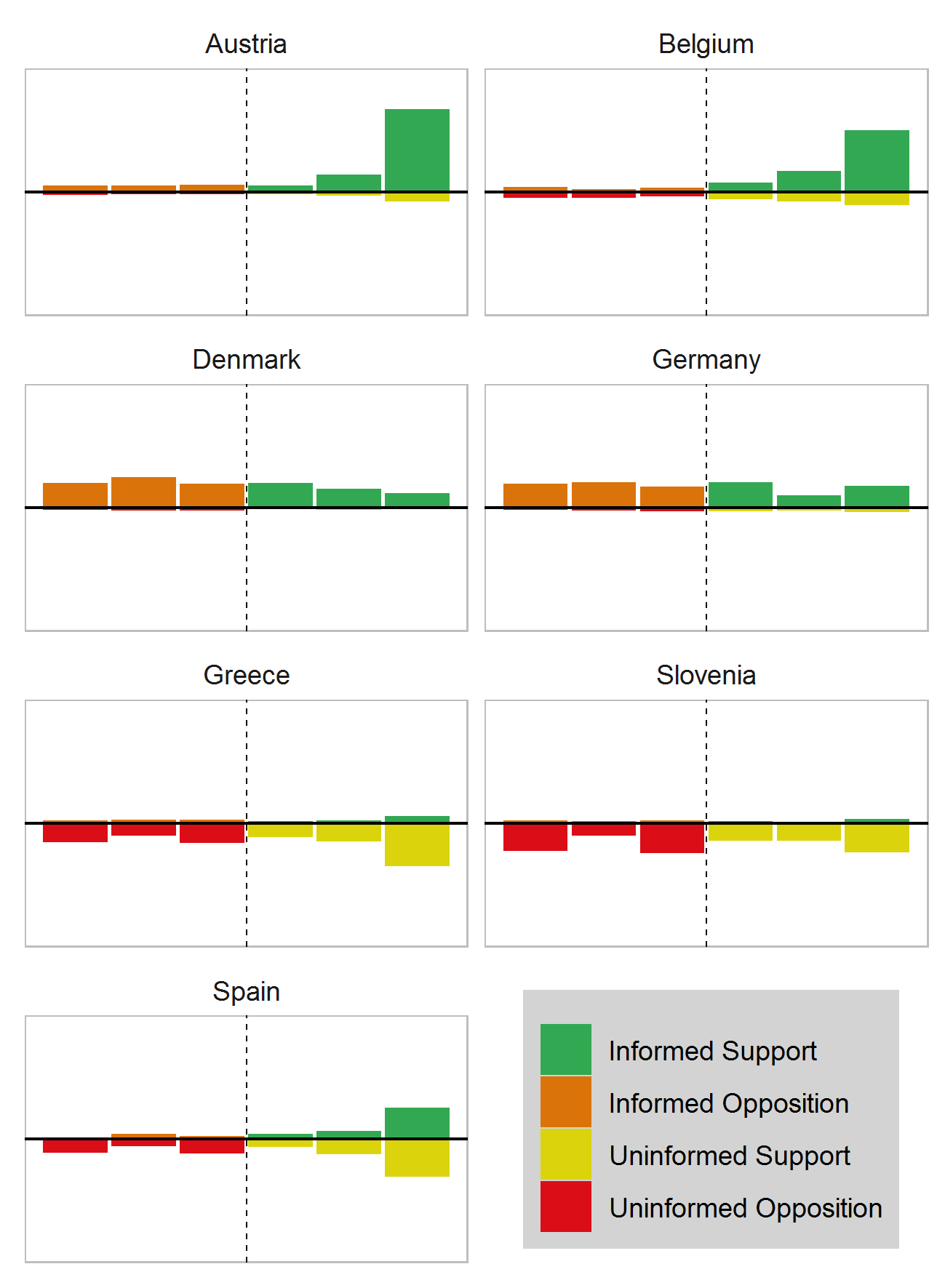


*Note: Fig A* displays the degree of support (x-axis), against knowledge (y-axis) for each country. In the top right quadrant (*Level A*), we chart the frequency of *informed support* –the best-case scenario. The remaining three quadrants display *informed opposition* (Level B_1_, top left), *misinformed support* (Level B_2_, bottom right) and *misinformed opposition* (Level C, bottom left). Bivariate histograms reflecting the national frequency of different scenarios based on the relation of knowledge and support of the consent policy in place. The x-axis displays the number of individuals reporting each level of opposition or support of the system in place, from 1 (leftmost-higher opposition) to 6 (rightmost-higher support). The y-axis displays the number of informed (above axis) and uninformed (below axis) participants. In Austria and Belgium, the vast majority of participants both know and support the policy in place (Level A), and those who are more supportive are also more knowledgeable. In Denmark and Germany, the vast majority of participants know the policy in place, but they are divided between those who support it (Level A) and those who don’t (Level B_1_). In Greece and Slovenia, the majority of participants ignore the policy actually in place in their country, and they are divided between those who support it (Level B_2_) and those who don’t (Level C). In Spain, a majority supports the policy in place but are divided between those who know the policy (Level A) and those who don’t (Level B_2_); and among those who are opposed to the policy, most are also uninformed (Level C). While *Fig A* considers the two variables (knowledge and support) simultaneously (i.e. individuals who both know *and* support (A), or don’t know *and* support (B2), etc.), *Fig 3* considers the two variables as independent from each other. Furthermore, while *Fig A* shows for each scenario how many students populate that scenario, thus classifying each country’s sample into four groups of students, *Fig 3* displays the overall distribution of the entire sample in a given country, thus situating the whole population, overall, in one of the four scenarios. It is also worth noting that the vertical axis in *Fig A* represents both the knowledge (those who know are above, those who don’t are below) and the level of support (per the height of the bars).

1. **Fig B. *Health Governance Indicator II: Fulfilment/contravention of unexpressed preferences and policy awareness***


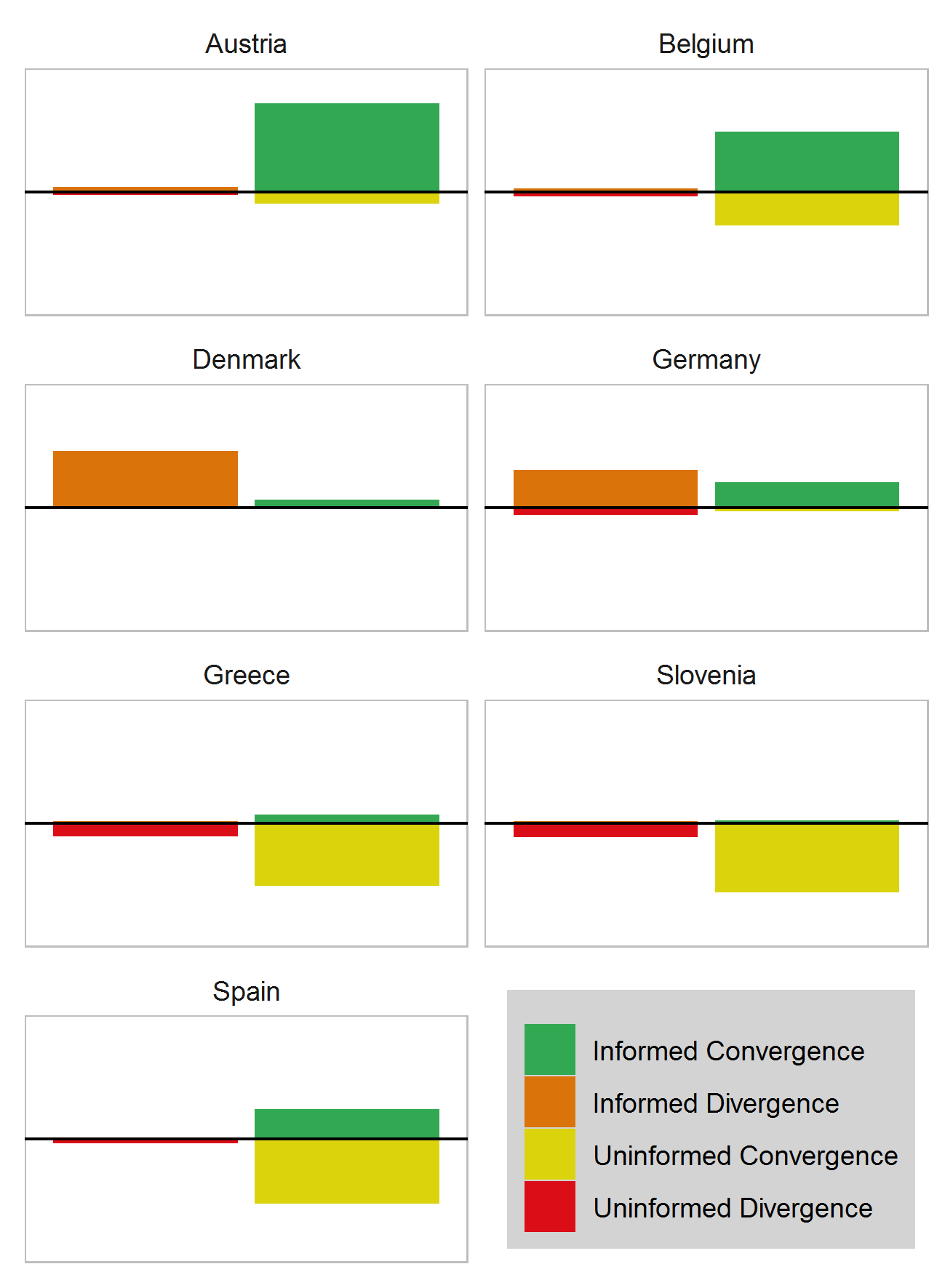


*Note: Fig B* displays *knowledge of the policy* against *fulfilment of non-expressed preferences* for each country. The top-right quadrant shows the proportion of individuals who both know the policy and whose non-expressed wishes would be fulfilled (informed convergence). The top-left quadrant shows the proportion of individuals who know the policy but whose non-expressed wishes would not be fulfilled (informed divergence). The lower-right quadrant shows the proportion of individuals who are unaware of the policy and whose non-expressed preferences would nevertheless be fulfilled (uninformed convergence). Finally, the lower-left quadrant shows the proportion of individuals who are unaware of the policy and whose noon-expressed preferences would be contravened (uninformed divergence). Bivariate histograms reflecting the national frequency of different scenarios based on the relation between knowledge (on the y-axis) and convergence with the consent policy in place (on the x-axis). In Austria and Belgium, the vast majority of participants both know and converge with the policy in place (Level A). In Denmark and Germany, the vast majority of participants know the policy in place, but diverge from it (Level B_1_). In Greece and Slovenia, the majority of participants ignore the policy actually in place in their country, but tend to converge with it (Level B_2_). In Spain, a majority converges with the policy in place but are divided between those who know the policy (Level A) and those who don’t (Level B_2_).

1. **Table 1. Sampling frame. (Overview of students who may have received information about the survey)**

| **Country and City of data collection** | **Year** | **Number of students who had potential access to the survey.**  **Medicine and Health Sciences** | **% of women** | **Number of students who had potential access to the survey.**  **Humanities/Social Sciences, Social Work/Social Management** | **% of women** |
| --- | --- | --- | --- | --- | --- |
| **Austria**  (Innsbruck & Hall in Tirol)^[[1]](#footnote-1)^ | 2019 | 2026 (MUI)  1700 (UMIT) | 54 % (MUI)  54 % (UMIT) | 4377 (LFUI)  260 (MCI) | 52 % (LFUI)  52 % (MCI) |
| **Belgium**  (Antwerpen & Ghent)^[[2]](#footnote-2)^ | 2019 | 2009 | 61% | 5081 | 65% |
| **Denmark**  (Copenhague)^[[3]](#footnote-3)^ | 2018-2019 | 402 | 72% | 405 | 57% |
| **Germany**  (Göttingen)^[[4]](#footnote-4)^ | 2018-2019 | 3741 | 65% | 8411 | 62% |
| **Greece**  (Thessaloniki)^[[5]](#footnote-5)^ | 2018-2019 | 1680 | 61% | 610 (IHU) | 39% |
| **Slovenia**  (Ljubljana) | 2018-2019 | N/A | N/A | N/A | N/A |
| **Spain**  (Granada)^[[6]](#footnote-6)^ | 2018-2019 | 1349 | 70% | 2569 | 57% |

1. **Participant Information Sheet**

**Information about voluntary participation in student survey about**

**Knowledge and attitudes about donation and transplantation of organs/body parts (ISSATO)**

# Participant Information Sheet

**Dear participant,**

thank you for your interest in our study.

- We would like to invite you to take part in our research study to see what people think about the donation and transplantation of organs and body parts.
- Before you decide whether you would like to take part it is important for you to understand why the research is being done and what it would involve for you.

**What is the purpose of the study?**

- Several European countries recently changed or considered to change their models of organ procurement and systems of consent for organ donation.
- This is an international quantitative survey of students from different fields of health sciences and social sciences/humanities to investigate their knowledge and attitudes towards different models of consent for postmortem organ donation in each country.
- The purpose of this study is to explore the perspectives and opinions of students about these developments and to increase the understanding of the social and ethical issues involved.

**Who can take part?**

- Anyone who is 18 years old or older and in his or her second year onwards study year at the university and study health sciences and social sciences/humanities is able to take part to our survey.

**Do I have to take part?**

- **No**. It is up to you to decide if you want to take part. If you do decide to take part you will be free to withdraw from the study at any time, without giving a reason.

**What will happen to me if I take part?**

- If you agree to take part in the study you will be asked at the end of this information shed to click to a box to start with the survey. The survey will take approximately 20 minutes.
- The survey is collecting all data anonymously

**What are the possible benefits of taking part?**

- As part of this study, you can win one of a 4 coupons up to 25 euros. If you would like to participate to the raffle, you can enter your e-mail address in the address field provided **after completing the survey**. The raffle will take place after completion of the survey. The personal data collect for this raffle will be collected separately from the survey data.
- Your contribution to the survey will allow the researchers to have a better understanding of public perceptions of the social and ethical issues associated with recent developments in organ transplantation, and may inform policy making in this area.

**Will my taking part in this study be kept confidential?**

- Yes. Any information you provide will be kept confidential. The survey data will be kept securely at the German University data management (GWDG) company. The survey data cannot be link to your email-address in case you take part in the raffle. All emails will be destroyed after the raffle has taken place (approx. 3 month after end of data collection)

**By clicking the box, you agree with the conditions outlined here and start the survey. You can print and keep this sheet of information for your records**

1. **Survey instrument. *International Student Survey about Knowledge and Attitudes about Donation and Transplantation of Organs/Body Parts (ISSATO)***

**International Student Survey about**

**Knowledge and Attitudes about Donation and Transplantation of Organs/Body Parts (ISSATO)**

This survey examines what European university students think about organ donation and transplantation. The survey will be undertaken in Belgium, Denmark, Germany, Greece, Israel, the Netherlands, Romania, Slovenia, Spain and England. It is the first comparative survey of organ donation and transplantation with students in Europe and we very much appreciate your participation.

The survey will take about 15-20 minutes.

**First we would like to ask about your prior experiences regarding organ donation and transplantation.**

**1. Is there anyone in your circle of family and friends who has donated an organ after death?**

Yes 🞎 No 🞎 Don’t know 🞎

**2. Is there anyone in your circle of family or friends who has received an organ?**

Yes 🞎 No 🞎 Don’t know 🞎

1. **Is there anyone in your circle of family or friends with a serious chronic organ disease**

**who might be in need of an organ transplant?**

Yes 🞎 No 🞎 Don’t know 🞎

**What do you know about the legal regulation of organ donation in your country?**

1. **There exist different legal systems for how citizens consent to the donation/procurement of their organs after death. Please indicate, which legal system is currently in place in your country.** *(Please give* ***one*** *answer only)*
2. Informed consent / opt-in system (explicit expressed wish) 🞎
3. Presumed consent / opt-out system (not refused during lifetime) 🞎
4. Other 🞎 *(please state)* ____________
5. I don’t know 🞎
6. **What do you know about the legal procedures to express one’s personal choice for or against donation in your country?** *(Please respond to* ***each*** *of the following statements)*
7. In my country there are official donor cards where I can express the wish

to donate or to reject organ donation.

Yes 🞎 No 🞎 Don’t know 🞎

1. In my country there is an official donor registry where I can express my

wish to donate organs.

Yes 🞎 No 🞎 Don’t know 🞎

1. In my country there is an official donor registry where I can express the wish

to donate or to reject organ donation.

Yes 🞎 No 🞎 Don’t know 🞎

1. In my country there is an official refusal registry where I can express my wish

not to donate organs.

Yes 🞎 No 🞎 Don’t know 🞎

1. In my country, there exists no possibility to express my personal wishes

about organ donation.

Yes 🞎 No 🞎 Don’t know 🞎

1. In my country I have to communicate my preferences to my family because they will be consulted about organ donation.

Yes 🞎 No 🞎 Don’t know 🞎

1. **According to the law in YOUR country, when the deceased person HAD expressed a preference regarding organ donation, what is the role of the family?** (Please give **one** answer only)

The family is not consulted at all about organ donation (no role) 🞎

The family is only asked to communicate the updated preferences of the deceased

in case these had recently changed 🞎

The family can veto organ donation when the deceased had consented to organ procurement 🞎

The family can authorise organ donation when the deceased had refused organ procurement 🞎

The family can both, veto and authorise organ donation 🞎

Don’t know 🞎

1. **According to the law in YOUR country, when the deceased person HAD NOT expressed a preference regarding organ donation, what is the role of the family?** (Please give **one** answer only!)

The medical team decides alone, the family is not consulted (but the team may prevent organ

procurement if there is evidence of family distress) 🞎

The family can oppose organ donation 🞎

The family can authorize organ donation 🞎

Don’t know 🞎

1. **There exists in Europe different regulations governing when organs can be procured after death. What type of death is allowed for the procurement of organs in your country?** (Please give **one** answer only)
2. Only donation after the irreversible cessation of the functions of the brain (in some countries brain death means brain stem death) 🞎

Only donation after the irreversible cessation of circulation 🞎

Both donation after the irreversible cessation of the functions of the brain, and donation after the irreversible cessation of circulation 🞎

Don’t know 🞎

**The following questions ask about your personal opinion towards organ donation and how it might be regulated.**

**9. Would you donate your organs after death**?

Yes 🞎 No 🞎 Don’t know 🞎

1. **Have you explicitly expressed your preference about organ donation and if so, how:** *(Please respond to* ***each*** *of the following statements)*
2. By a donor card

Yes 🞎 No 🞎 Don’t want to tell 🞎

1. By a public/legal registry

Yes 🞎 No 🞎 Don’t want to tell 🞎

1. By informing my close relatives about my preferences

Yes 🞎 No 🞎 Don’t want to tell 🞎

1. By other means 🞎 *(please state)* ____________

***If you answered ‘yes’ to any of the statements in Question 10, please proceed to Question 12.***

**If you answered ‘no’ to all of the statements in Question 10, please answer question 11**

1. **Which of these reasons best explain why you have not expressed your wishes?** *(Please fill out* ***each*** *line)*
2. I haven’t yet thought about the issue

Yes 🞎 No 🞎 Don’t know 🞎

1. I’m undecided whether I want to donate

Yes 🞎 No 🞎 Don’t know 🞎

1. I’m afraid that physicians might be more interested in my organs than in saving my live

Yes 🞎 No 🞎 Don’t know 🞎

1. I haven’t had the opportunity to express my wish

Yes 🞎 No 🞎 Don’t know 🞎

1. In my country I don’t need to be registered to become an organ donor

Yes 🞎 No 🞎 Don’t know 🞎

1. I don’t know how to/where I can make my wish clear concerning organ donation

Yes 🞎 No 🞎 Don’t know 🞎

1. **What do you know about the attitudes towards organ donation in your social environment?** (Please give **one** answer only)

My social environment is in favour of organ donation 🞎

My social environment is against organ donation 🞎

I do not know 🞎

1. **What do you believe are the attitudes towards organ donation among the majority of the population in your country?** (Please give **one** answer only)

The majority of the population is for organ donation 🞎

The majority of the population is against organ donation 🞎

I do not know 🞎


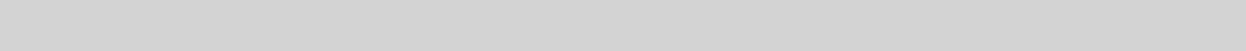


**The following questions ask your opinion about which organs or body parts should be considered for deceased donation?**

|  |  |  |  |  |  |
| --- | --- | --- | --- | --- | --- |
|  |  |  |  |  |  |

1. **Which organs and body parts you would like to donate after death?** *(Please tick one answer in* ***each*** *line)*

Fully Accept

Fully Reject

|  | 1 | 2 | 3 | 4 | 5 | 6 |
| --- | --- | --- | --- | --- | --- | --- |
| Bone |  |  |  |  |  |  |
| Cornea |  |  |  |  |  |  |
| Face |  |  |  |  |  |  |
| Genitalia |  |  |  |  |  |  |
| Heart |  |  |  |  |  |  |
| Individual limbs (e.g. a hand, arm, or leg) |  |  |  |  |  |  |
| Intestine |  |  |  |  |  |  |
| Kidney |  |  |  |  |  |  |
| Larger area of skin |  |  |  |  |  |  |
| Liver |  |  |  |  |  |  |
| Lungs |  |  |  |  |  |  |
| Pancreas |  |  |  |  |  |  |
| Uterus |  |  |  |  |  |  |
| None |  |  |  |  |  |  |

1. **In many countries, organs are procured after brain death. Which of these statements do you agree with?** *(Please tick one answer in* ***each*** *line)*

Fully Accept

Fully Reject

|  | 1 | 2 | 3 | 4 | 5 | 6 |
| --- | --- | --- | --- | --- | --- | --- |
| When the brain is totally and irreversibly  damaged and completely stopped functioning,  but the person is still on mechanical ventilation, the  he/she is DEAD |  |  |  |  |  |  |
| When the brain is totally and irreversibly  damaged and completely stopped functioning,  although the person is on mechanical ventilation, he/she is ALIVE |  |  |  |  |  |  |
| When those regions of the brain connected with  personality, thinking, and speaking are totally  and irreversibly damaged and have completely  stopped functioning, the individual is dead  although he/she is on mechanical ventilation |  |  |  |  |  |  |
| The individual is dead when he or she is  irreversibly unconscious and will never wake up  again, even if the body is kept by technical  means functioning |  |  |  |  |  |  |

1. **Why would you decide in favour of donating an organ after death?** *(Please tick one answer in* ***each*** *line)*
2. I want to help other people

Yes 🞎 No 🞎 Don’t know 🞎

1. Responsibility to society

Yes 🞎 No 🞎 Don’t know 🞎

1. Moral sense of duty

Yes 🞎 No 🞎 Don’t know 🞎

1. Because it would get special social recognition

Yes 🞎 No 🞎 Don’t know 🞎

1. It is a medical advance that is essential

Yes 🞎 No 🞎 Don’t know 🞎

1. My body is useless after I die

Yes 🞎 No 🞎 Don’t know 🞎

1. I also want to get an organ if I need one (reciprocity)

Yes 🞎 No 🞎 Don’t know 🞎

1. Giving sense to my own death

Yes 🞎 No 🞎 Don’t know 🞎

**17. Why would you potentially decide against donating an organ after death?**

*(Please tick one answer in* ***each*** *line)*

1. It constitutes an invasion of my bodily integrity

Yes 🞎 No 🞎 Don’t know 🞎

1. Fear that the recipient’s body will reject my organ.

Yes 🞎 No 🞎 Don’t know 🞎

1. It harms my soul’s integrity

Yes 🞎 No 🞎 Don’t know 🞎

1. Lack of trust in physicians / medical care

Yes 🞎 No 🞎 Don’t know 🞎

1. Distress for my family

Yes 🞎 No 🞎 Don’t know 🞎

1. Concern that there is no proper end-of-life care

Yes 🞎 No 🞎 Don’t know 🞎

1. Fear of what happens during organ procurement

Yes 🞎 No 🞎 Don’t know 🞎

1. No right to decide to whom my organs go

Yes 🞎 No 🞎 Don’t know 🞎

1. I do not believe donors are really dead

Yes 🞎 No 🞎 Don’t know 🞎

1. None of the above 🞎 *(please explain why)* ___________

**The following questions are about receiving an organ from a deceased donor**

**18**. **In the case that you were in need of an organ/body parts, which of the following would you accept?** *(Please tick one answer in* ***each*** *line)*

|  | 1 | 2 | 3 | 4 | 5 | 6 |
| --- | --- | --- | --- | --- | --- | --- |
| Bone |  |  |  |  |  |  |
| Cornea |  |  |  |  |  |  |
| Face |  |  |  |  |  |  |
| Genitalia |  |  |  |  |  |  |
| Heart |  |  |  |  |  |  |
| Individual limbs (e.g. a hand, arm, or leg) |  |  |  |  |  |  |
| Intestine |  |  |  |  |  |  |
| Kidney |  |  |  |  |  |  |
| Larger area of skin |  |  |  |  |  |  |
| Liver |  |  |  |  |  |  |
| Lungs |  |  |  |  |  |  |
| Pancreas |  |  |  |  |  |  |
| Uterus |  |  |  |  |  |  |
| None |  |  |  |  |  |  |

Fully Accept

Fully Reject

1. **If all of the following options were equally available and equally effective, which one would you prefer?** *(Please tick one answer in* ***each*** *line)*

|  | Yes | No | Don’t Know |
| --- | --- | --- | --- |
| The organ of a brain dead donor |  |  |  |
| The organ of a person with no heart activity (known as non-heart beating donor. Not possible for the heart) |  |  |  |
| An organ of an animal (e.g. pig, monkey) (xenotransplant) |  |  |  |
| An organ grown from stem cells |  |  |  |
| A 3D printed organ |  |  |  |
| An artificial/mechanical organ |  |  |  |
| I don’t care as long as I recover |  |  |  |
| I oppose any kind of organ transplantation |  |  |  |

**The following questions concern your image of the human body.**

1. **Which of the following statements on the human body would you agree with?** *(Please tick one answer in* ***each*** *line)*

|  | Yes | No | Don’t Know |
| --- | --- | --- | --- |
| The human body is comparable to a machine; where all individual parts can simply be replaced |  |  |  |
| The human body is more than the sum of its parts; thus, individual parts cannot always just be replaced |  |  |  |
| Determine a person’s individuality and uniqueness and should not be transplanted |  |  |  |

1. **Which of the following organs is essential for a person’s identity/personality according to your opinion?**

*(Please tick one answer in* ***each*** *line)*

|  | Yes | No | Don’t Know |
| --- | --- | --- | --- |
| Brain |  |  |  |
| Heart |  |  |  |
| Kidney |  |  |  |
| Liver |  |  |  |
| Lung |  |  |  |
| None of them |  |  |  |

Other 🞎 *(please explain)* ___________

**Please answer a few questions about issues discussed in public discourse and politics. We would like to know your personal views on these issues.**

1. **Which of the statements on organ donation and transplantation do you agree with?** *(Please tick one answer in* ***each*** *line)*

Fully Agree

Never Agree

|  | 1 | 2 | 3 | 4 | 5 | 6 |
| --- | --- | --- | --- | --- | --- | --- |
| There is a shortage of organs |  |  |  |  |  |  |
| The use of brain-dead person as organ donors is morally problematic |  |  |  |  |  |  |
| The use of non-heart-beating persons as organ donors is morally problematic |  |  |  |  |  |  |
| The regulation concerning organ donation is morally problematic |  |  |  |  |  |  |
| Organ donation can give comfort to mourning family |  |  |  |  |  |  |
| The allocation of organs is unjust |  |  |  |  |  |  |
| Organ donation gives death a meaning |  |  |  |  |  |  |
| The allocation of organs is not transparent |  |  |  |  |  |  |
| Organ donation is a citizen’s duty |  |  |  |  |  |  |

1. **Regardless of the legal system of consent for organ procurement in your country, which system do you prefer?** *(Please tick one answer in* ***each*** *line)*

|  | 1 | 2 | 3 | 4 | 5 | 6 |
| --- | --- | --- | --- | --- | --- | --- |
| A system where I become a donor unless I have expressed a refusal (presumed consent) |  |  |  |  |  |  |
| A system where I’m a donor only when I have explicitly expressed a willingness to donate (informed consent) |  |  |  |  |  |  |
| A system where organs are mandatorily procured from the deceased, regardless of their preferences or those of their relatives |  |  |  |  |  |  |
| A system where I’m legally required to express my preferences about organ procurement (e.g. on my driver license or on my passport) |  |  |  |  |  |  |

Fully Disagree

Fully Agree

1. **Regardless of the legal system of consent for organ procurement in your country, which role should families have when the preferences of the deceased ARE known?**

(*Please give* ***one*** *answer only)*

A system where the family is not consulted about organ procurement 🞎

A system where the family is only asked to communicate the updated

preferences of the deceased in case these had recently changed 🞎

A system where the family can impede organ procurement when

the deceased had consented organ procurement 🞎

A system where the family can authorize organ

procurement when the deceased had refused organ procurement 🞎

A system where the preferences of the family are followed regardless

of the preferences of the deceased 🞎

Don’t know 🞎

1. **Regardless of the legal system of the country you live in, which role should families have when the preferences of the deceased ARE NOT known?**

*(Please give* ***one*** *answer only)*

A system where the family is not consulted about organ procurement. 🞎

The system at work prevails (e.g. organs are procured in presumed

consent, and organs are not procured in explicit consent) 🞎

A system where the medical team decides (e.g. they may prevent

organ procurement because of evidence of family distress) 🞎

A system where the family can oppose organ donation 🞎

A system where the family can authorize organ donation 🞎

Don’t know 🞎

1. **It is discussed whether we should have a harmonized EU-Regulation ensuring the same regulation in each European country. Please indicate your level of agreement with each statement.**

*(Please tick one answer in* ***each*** *line)*

|  | 1 | 2 | 3 | 4 | 5 | 6 |
| --- | --- | --- | --- | --- | --- | --- |
| All countries should have an opt-out system |  |  |  |  |  |  |
| All countries should have an opt-in system |  |  |  |  |  |  |
| In all countries, the preferences of the family should always  be followed in decision making related to organ donation |  |  |  |  |  |  |
| In all countries, the preferences of the family should always  be followed in decision making related to organ donation  only in cases where there is no explicitly expressed  preference by the deceased |  |  |  |  |  |  |
| Each country should develop their own regulation on the  consent system for deceased donation |  |  |  |  |  |  |
| The public in each country should be involved in discussions about legal changes on the consent system for organ  donation |  |  |  |  |  |  |

Fully Agree

Never Agree

1. **Do you feel sufficiently informed about the topic of organ donation?**

Yes 🞎 No 🞎 Don’t know 🞎

1. **According to your preferences, who should provide more information about organ donation?** *(Please tick one answer in* ***each*** *line)*
2. The State (e.g. ministries, the government) at official websites

Yes 🞎 No 🞎 Don’t know 🞎

1. Transplant surgeons

Yes 🞎 No 🞎 Don’t know 🞎

1. Church/religious groups

Yes 🞎 No 🞎 Don’t know 🞎

1. Media

Yes 🞎 No 🞎 Don’t know 🞎

1. Public school

Yes 🞎 No 🞎 Don’t know 🞎

1. Patient support groups

Yes 🞎 No 🞎 Don’t know 🞎

1. Others *(please state)* ___________

**We would now like to know about your degree course:**

**29. What is your main area of study?** *(Please tick only* ***one*** *option*)

Anthropology 🞎

Economics 🞎

Health Science 🞎

Humanities 🞎

Medicine 🞎

Nursing 🞎

Philosophy 🞎

Public Health 🞎

Social Work 🞎

Sociology 🞎

Other 🞎 *(please state)* ___________

1. **Which year you are studying in your current, main topic?** *(Please tick* only **one** *option*)

2^nd^ year (terms 4-6 or semesters 3 or 4) 🞎

3^rd^ Year (terms 7-9 or semesters 5 or 6) 🞎

4^th^ year (semesters 7 or 8) 🞎

5^th^ year (semesters 9 or 10) 🞎

**Finally there are a few questions about yourself and your personal circumstances.**

1. **I am…**

Male 🞎 Female 🞎 Prefer not to say 🞎

1. **How old are you?**

Younger than 20 🞎 20 to 24 🞎

25 to 29 🞎 30 to 39 🞎

40 to 49 🞎 50 to 59 🞎

60 to 69 🞎 70 + 🞎

1. **Would you describe yourself as a religious person?**

Spiritual 🞎

Very religious 🞎

Religious 🞎

Somewhat religious 🞎

Not religious 🞎

**34. If you are religious, what is your religion?**

Buddhist 🞎 Sikh 🞎

Greek Orthodox 🞎 None 🞎

Hindu 🞎 Other 🞎*(please state)* ___________

Jewish 🞎

Muslim 🞎

Protestant 🞎

Roman Catholic 🞎

Russian Orthodox 🞎

| **35. I found understanding this questionnaire…** |  |
| --- | --- |
|  |  |
| Very easy 🞎 | Difficult 🞎 |
| Easy 🞎 | Impossible 🞎 |
| **36. I found the topic of the questionnaire…** |  |
| Very interesting 🞎 | Not very interesting 🞎 |
| Interesting 🞎 | Not interesting 🞎 |
|  |  |

**Thank you for completing the questionnaire!**

If you want to take part in the raffle, please click to leave your contact data here. An e-mail address will do. The contact information will be stored and processed separately. For this purpose, you will be redirected to another website so it is not possible to trace back a single person.

1. Austria: recruitment via university and faculty mailing lists, as well as promotion in courses by lecturers, at Medical University Innsbruck (MUI), the health & life sciences university Hall in Tirol (UMIT), University of Innsbruck (LFUI), and Management Center Innsbruck (MCI). [↑](#footnote-ref-1)
2. Belgium: recruitment via faculty mailing lists, public poster campaigns, and student involvement in specific courses. [↑](#footnote-ref-2)
3. Denmark: Recruitment via promotion in courses by Jensen, followed by emails with survey link to students via online course platforms [↑](#footnote-ref-3)
4. Germany: Recruitment via faculty mailing-lists, public poster campaigns, flyer, newsletter. [↑](#footnote-ref-4)
5. Greece: Recruitment via flyers and promotion in courses by lecturers, at Aristotle University of Thessaloniki (AUTH: medicine) and International Hellenic University (IHU: nursing; and also non-medical or non-health sciences students). [↑](#footnote-ref-5)
6. Spain: Recruitment via faculty mailing-lists, public poster campaigns, University of Granada website, as well as promotion in courses by lecturers. [↑](#footnote-ref-6)
